# Electrocardiographic Predictors of Early Recurrence of Atrial Fibrillation After Electrical Cardioversion

**DOI:** 10.1111/anec.70225

**Published:** 2026-08-28

**Authors:** Keisuke Yonezu, Tetsuji Shinohara, Hirochika Yamasaki, Kei Hirota, Hidekazu Kondo, Akira Fukui, Hidefumi Akioka, Yasushi Teshima, Naohiko Takahashi

**Affiliations:** ^1^ Department of Cardiology and Clinical Examination, Faculty of Medicine Oita University Oita Japan

**Keywords:** atrial fibrillation, early recurrence, electrical cardioversion, fragmented QRS, notched P‐wave

## Abstract

**Background:**

Although electrical cardioversion (ECV) effectively restores sinus rhythm in patients with persistent atrial fibrillation (AF), early recurrence after successful ECV remains common in clinical practice. We investigated whether simple 12‐lead electrocardiographic markers and a composite ECG score could predict early AF recurrence.

**Methods:**

We retrospectively analyzed 195 patients with persistent AF who underwent successful ECV. Electrocardiograms recorded immediately after ECV were evaluated for notched P‐wave, fragmented QRS (fQRS), and early repolarization pattern (ERP). The ECG composite score was defined as the sum of these markers (0–3). Early AF recurrence was defined as electrocardiographically documented recurrence within 10 days after ECV. Multivariable logistic regression analysis was performed.

**Results:**

Early AF recurrence occurred in 100 patients (51.3%). AF duration was longer in patients with recurrence (12 [3–57] vs. 5 [2–15] months, *p* < 0.001). Notched P‐wave (76.0% vs. 24.2%, *p* < 0.001), fQRS (74.0% vs. 57.9%, *p* = 0.017), and ERP (41.0% vs. 24.2%, *p* = 0.015) were more frequent in patients with recurrence. In multivariable analysis, notched P‐wave (OR 11.80), fQRS (OR 2.34), and ERP (OR 3.54) independently predicted recurrence (all *p* < 0.05). The ECG composite score was also independently associated with recurrence (OR 4.91 per 1‐point increase, *p* < 0.001), with a stepwise increase in recurrence across score categories.

**Conclusions:**

Simple 12‐lead ECG markers and a composite ECG score may help predict early AF recurrence and guide rhythm‐control strategies after ECV in patients with persistent AF.

## Graphical abstract

A simple ECG composite score predicts early AF recurrence after electrical cardioversion and may help guide rhythm‐control strategies.

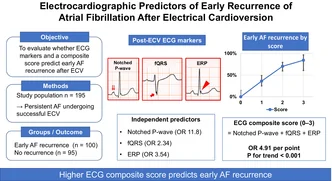

## 1 Introduction

Atrial fibrillation (AF) is the most common sustained arrhythmia in clinical practice and is associated with an increased risk of stroke, heart failure, sudden death, and overall morbidity (Benjamin et al. 1998). Recent advances in AF management have highlighted the clinical benefit of rhythm‐control therapy, particularly early rhythm control (Gu et al. 2024; Kirchhof et al. 2020). Electrical cardioversion (ECV) and pharmacological cardioversion are established strategies for restoring sinus rhythm. Compared with pharmacological cardioversion, ECV has the advantage of avoiding drug‐related adverse effects such as proarrhythmia, conduction disturbances, and worsening heart failure. In addition, ECV can rapidly restore sinus rhythm in more than 90% of patients (Berry et al. 2001; Pisters et al. 2012; Toso et al. 2012). However, maintenance of sinus rhythm after successful ECV remains challenging, with nearly half of patients experiencing AF recurrence within 1 year (Toso et al. 2012), and many recurrences occurring within the first week.

Several clinical and echocardiographic factors have been reported to predict AF recurrence after ECV. A longer AF duration, particularly ≥ 6 months, has been associated with a higher recurrence risk (Toso et al. 2012). In addition, left ventricular systolic dysfunction and left atrial enlargement have been identified as predictors of recurrence (Marchese et al. 2011). Regarding electrocardiographic markers, P‐wave duration and P‐wave dispersion have also been linked to AF recurrence after ECV (Fujimoto et al. 2018). The notched P‐wave reflects atrial conduction delay (Dilaveris et al. 1998) and has been associated with AF occurrence (Huang et al. 2020; Okuyama et al. 2024). Furthermore, fragmented QRS (fQRS) (Yesin et al. 2018) and early repolarization pattern (ERP) (Hasegawa et al. 2019) on 12‐lead ECG have also been reported to be associated with AF development. However, the predictive value of these ECG markers for early AF recurrence after ECV has not been fully investigated.

Identifying patients at high risk of early recurrence may help optimize patient selection for ECV and subsequent rhythm‐control strategies. Therefore, we aimed to evaluate the usefulness of simple 12‐lead ECG parameters, including notched P‐wave, fQRS, and ERP, for predicting early AF recurrence after ECV in patients with persistent AF.

## 2 Methods

### 2.1 Study Population

**FIGURE 1:**
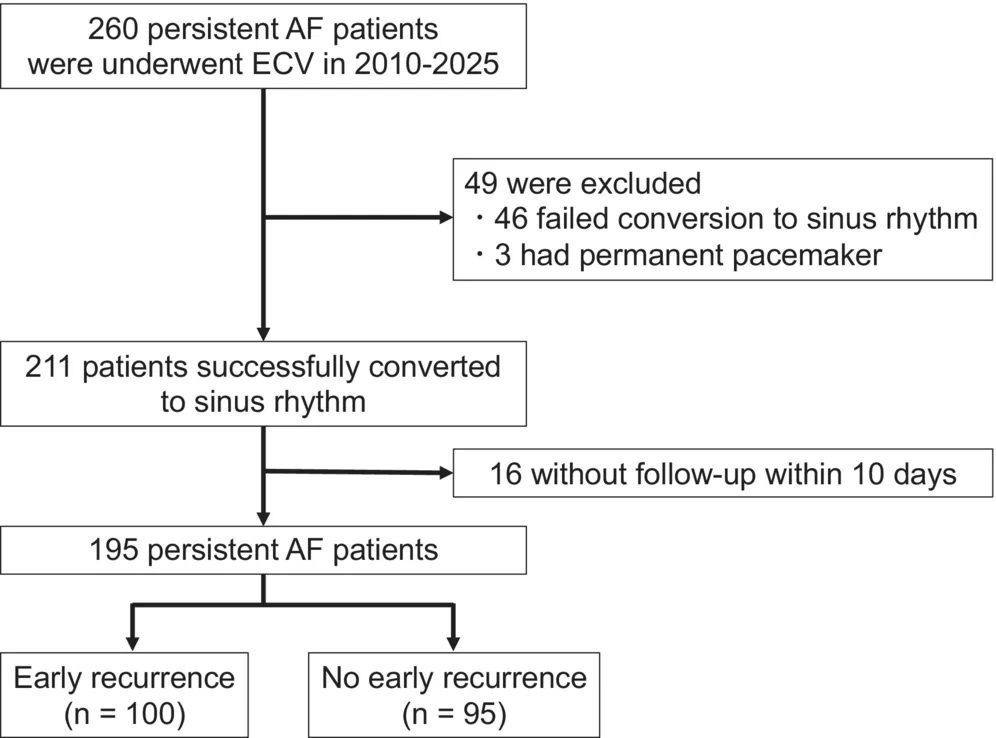
Study flow chart. A total of 260 patients with persistent atrial fibrillation (AF) underwent electrical cardioversion (ECV) between January 2010 and December 2025. Among these, 49 patients were excluded because of prior pacemaker implantation (*n* = 3) or failure to restore sinus rhythm after ECV (*n* = 46). Among the 211 patients in whom sinus rhythm was successfully restored, 16 patients without follow‐up within 10 days were further excluded. The final study population consisted of 195 patients, including 100 patients with early AF recurrence and 95 patients without recurrence. AF, atrial fibrillation; ECV, electrical cardioversion.

This retrospective observational study included consecutive patients who underwent ECV for persistent AF at our institution between January 2010 and December 2025. Of the 260 patients screened, 49 were excluded because of failure to restore sinus rhythm after ECV (*n* = 46) or prior permanent pacemaker implantation (*n* = 3). Among the 211 patients in whom sinus rhythm was successfully restored, 16 patients without follow‐up within 10 days were further excluded. The final study population consisted of 195 patients (Figure 1).

This study was approved by the institutional review board of our institution (approval number: 1283) and was conducted in accordance with the Declaration of Helsinki. The requirement for written informed consent was waived because of the retrospective study design.

### 2.2 Clinical Course

Standard 12‐lead electrocardiograms were recorded immediately after successful ECV during sinus rhythm. After ECV, all patients were hospitalized overnight and monitored using continuous electrocardiographic telemetry until discharge on the following day. Patients were also instructed to perform self‐monitoring of their pulse after discharge and to contact the hospital if symptoms suggestive of AF recurrence occurred.

Follow‐up was performed 10 days after ECV at a scheduled outpatient visit, according to the routine early follow‐up schedule at our institution. AF recurrence was confirmed by electrocardiographic documentation during hospitalization or at the follow‐up visit. Early recurrence was defined as AF recurrence documented within 10 days after ECV.

### 2.3 Twelve‐Lead ECG Findings

**FIGURE 2:**
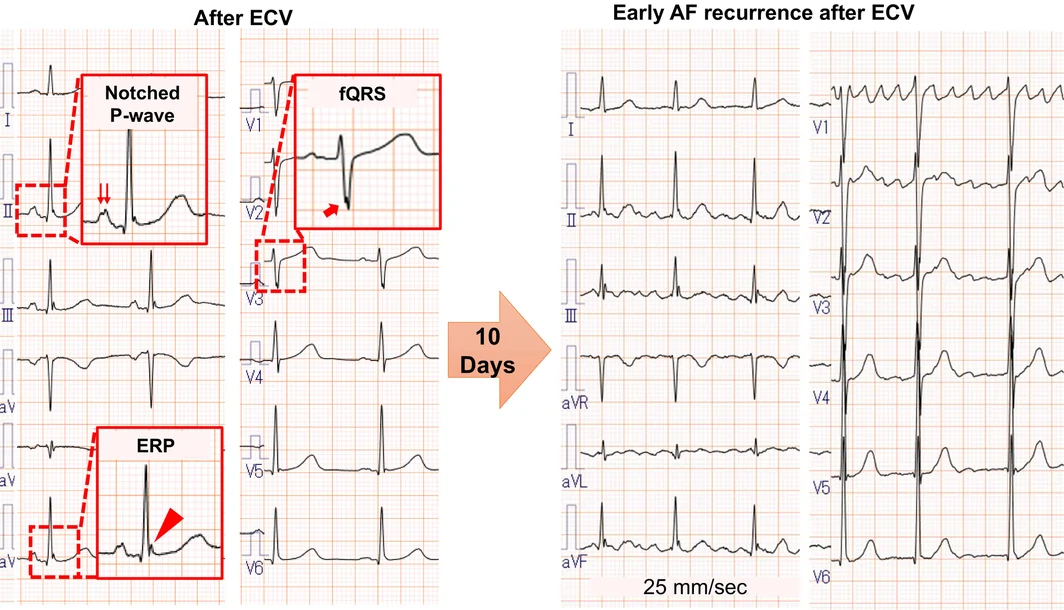
Representative electrocardiographic findings associated with early atrial fibrillation recurrence. Representative 12‐lead electrocardiograms demonstrating the three electrocardiographic markers associated with early atrial fibrillation (AF) recurrence after electrical cardioversion (ECV): (A) notched P‐wave in Lead II, (B) fragmented QRS (fQRS) in V_2_–V_3_, and (C) early repolarization pattern (ERP) with terminal QRS notching or slurring in the inferior (II, III, aV_F_) and lateral leads (V_5_–V_6_). AF, atrial fibrillation; ECV, electrical cardioversion; ERP, early repolarization pattern; fQRS, fragmented QRS.

A notched P‐wave was defined as an M‐shaped P‐wave in Lead II with a peak‐to‐peak interval ≥ 40 ms (Figure 2) (Yonezu et al. 2025). P‐terminal force in Lead V_1_ was calculated as the product of the duration of the 7terminal negative portion of the P‐wave (ms) and its amplitude (mV), expressed as an absolute value. The presence of fQRS was defined as the presence of additional spikes within the QRS complex in at least two consecutive leads with QRS duration < 120 ms according to established criteria (Das et al. 2006). ERP was defined as a notch or slur in the terminal part of the QRS complex with an amplitude ≥ 0.1 mV in at least two contiguous inferior (II, III, aVF) or lateral leads (I, aVL, V_4_–V_6_) (Macfarlane et al. 2015). All electrocardiograms were recorded using a standard 12‐lead system (Cardiofax, Fukuda Denshi, Tokyo, Japan) with a low‐pass filter setting of 150 Hz. ECG tracings were magnified to 400%, and all measurements were performed manually. The ECG composite score was defined as the sum of the presence of notched P‐wave, fQRS, and ERP, with 1 point assigned for each variable (range 0–3).

### 2.4 Reproducibility

To assess intraobserver reproducibility, the same observer reassessed the same electrocardiograms after a 1‐week interval. To assess interobserver reproducibility, a second blinded observer independently evaluated the same electrocardiograms without knowledge of the first observer's results or clinical outcomes. Cohen's *κ* values and overall agreement rates were calculated for notched P‐wave, fQRS, and ERP. Disagreements were resolved by consensus.

### 2.5 Statistical Analysis

Continuous variables are presented as median (interquartile range) and categorical variables as number (%). Normality was assessed using the Shapiro–Wilk test. Normally distributed continuous variables were compared using the unpaired Student's *t*‐test, whereas non‐normally distributed variables were compared using the Mann–Whitney *U* test. Categorical variables were compared using the chi‐square test or Fisher's exact test, as appropriate. Univariate logistic regression analysis was performed to identify variables associated with early AF recurrence within 10 days. Variables with *p* < 0.05 were entered into multivariable logistic regression models. Two models were constructed: Model 1 included individual ECG variables (notched P‐wave, fQRS, and ERP), whereas Model 2 included the ECG composite score instead of the individual ECG variables to avoid multicollinearity. Age and sex were included as clinically relevant covariates. Associations between ECG composite score categories (0–3) and early AF recurrence were evaluated using logistic regression, including tests for trend across ordered groups. Pairwise comparisons (1 vs. 2, 1 vs. 3, and 2 vs. 3) were also performed. As a supplementary analysis, a regression coefficient‐based weighted ECG score was created using the regression coefficients of the individual ECG predictors. The discriminative abilities of the equal‐weight ECG composite score and the weighted ECG score were evaluated using receiver operating characteristic curve analysis, and their areas under the curve (AUC) were descriptively compared. Optimal cutoff values were determined using the Youden index. Odds ratios (ORs) with 95% confidence intervals (CIs) were calculated. To reduce overfitting, the number of variables in multivariable models was limited according to the number of outcome events, and multicollinearity was assessed using variance inflation factors. Intra‐ and interobserver reproducibility for ECG interpretation was assessed using Cohen's *κ* values and overall agreement rates. A two‐sided *p* < 0.05 was considered statistically significant. All analyses were performed using JMP version 14.3.0 for Windows (SAS Institute, Cary, NC, USA).

## 3 Results

### 3.1 Patient Characteristics

Baseline characteristics of the study population are summarized in Table 1. Patients with early AF recurrence within 10 days had a significantly longer AF duration than those without recurrence (12 [3–57] vs. 5 [2–15] months, *p* = 0.0007) and a higher prevalence of AF duration ≥ 6 months (63.0% vs. 48.4%, *p* = 0.040). There were no significant differences in age, sex, body mass index, CHADS_2_ score, CHA_2_DS_2_‐VASc score, or heart rate before and after ECV between the two groups.

**TABLE 1:** Baseline characteristics according to atrial fibrillation recurrence within 10 days.

	Overall (*n* = 195)	Recurrence (+) (*n* = 100)	Recurrence (−) (*n* = 95)	*p*
Age, year	67 (60–74)	66 (59–73)	68 (60–74)	0.5360
Male sex, *n* (%)	153 (78.5%)	80 (80.0%)	73 (76.8%)	0.5920
BMI, kg/m^2^	23.9 (21.9–26.6)	24.4 (22.7–26.6)	23.9 (21.9–26.1)	0.4500
CHADS_2_ score, points	1 (1–2)	1 (1–2)	1 (1–2)	0.0926
CHA_2_DS_2_‐VASc score, points	2 (1–3)	2 (1–3)	2 (2–4)	0.1525
Heart failure, *n* (%)	38 (19.5%)	18 (18.0%)	20 (21.1%)	0.1042
Underlying cardiac disease, *n* (%)				0.2861
Ischemic heart disease	10 (5.1%)	4 (4.0%)	6 (6.3%)	
Hypertensive heart disease	1 (0.5%)	1 (1.0%)	0 (0%)	
Cardiomyopathy	8 (4.1%)	2 (2.0%)	6 (6.3%)	
Congenital heart disease	2 (1.0%)	0 (0%)	2 (2.1%)	
Arrhythmia‐induced cardiomyopathy	2 (1.0%)	1 (1.0%)	1 (1.1%)	
Valvular heart disease	11 (5.6%)	5 (5.0%)	6 (6.3%)	
AF duration, months	7 (2–48)	12 (3–57)	5 (2–15)	0.0007**
AF duration ≥ 6 months, *n* (%)	109 (55.9%)	63 (63.0%)	46 (48.4%)	0.0400*
Heart rate before ECV, bpm	82 (72–94)	81.5 (72–94)	83 (72–96)	0.4760
Heart rate after ECV, bpm	66 (60–73)	68 (60–76.8)	65 (59–73)	0.0620
12‐lead ECG findings				
PR interval, ms	191 (170–218)	195 (171–222)	188 (170–214)	0.5974
QRS duration, ms	101 (94–109)	101 (94–108.8)	101 (94–110)	0.4940
QT interval, ms	412 (385–440)	405.5 (385–428.8)	419 (389–451)	0.0310
QTc interval (Bazett), ms	438 (424–462)	437.5 (424–457)	439 (418–462)	0.9120
QTc interval (Fridericia), ms	428 (409–447)	428 (412–445.8)	428 (409–457)	0.4320
12‐lead ECG related to P‐wave				
Notched P‐wave, *n* (%)	99 (50.8%)	76 (76.0%)	23 (24.2%)	< 0.0001**
P‐wave duration II, ms	128 (116–140)	128 (116–140)	128 (114–136)	0.4510
P‐wave amplitude II, mV	0.11 (0.08–0.14)	0.11 (0.08–0.14)	0.11 (0.08–0.13)	0.6070
P‐wave duration V_1_, ms	124 (112–140)	124 (112–144)	124 (112–136)	0.7440
P‐wave negative amplitude V_1_, mV	0.08 (0.06–0.10)	0.08 (0.06–0.10)	0.08 (0.06–0.10)	0.7110
P‐terminal force, mV ms	99 (72–132)	102 (77–132)	96 (72–136)	0.6460
Fragmented QRS, *n* (%)	129 (66.2%)	74 (74.0%)	55 (57.9%)	0.0170*
Total number of leads with fQRS on standard 12‐lead ECG, *n*	2 (0–3)	2 (2–4)	2 (0–3)	0.0290*
ERP, *n* (%)	64 (32.8%)	41 (41.0%)	23 (24.2%)	0.0148*
Inferior, *n* (%)	36 (18.5%)	24 (24.0%)	12 (12.6%)	
Inferolateral, *n* (%)	10 (5.1%)	9 (9.0%)	1 (1.1%)	
Lateral, *n* (%)	18 (9.2%)	8 (8.0%)	10 (10.5%)	
ERP + fQRS, *n* (%)	36 (18.5%)	26 (26.0%)	10 (10.5%)	0.0058**
ERP + notched P‐wave, *n* (%)	34 (17.5%)	30 (30.0%)	4 (4.2%)	< 0.0001**
Notched P‐wave + fQRS, *n* (%)	73 (37.4%)	55 (55.0%)	18 (19.0%)	< 0.0001**
ECG composite score	1 (0–2)	2.0 (1.3–2.0)	1.0 (1.0–2.0)	< 0.0001**
Echocardiogram				
Left ventricular ejection fraction, %	59 (51–66)	59.1 (50.6–63.2)	59.6 (50.9–66.6)	0.3350
Left atrial diameter (LAD), mm	44 (40–48)	43.9 (40.4–48.4)	44.7 (40.5–47.7)	0.8060
Left atrial diameter indexed to body surface area (LADI), mm/m^2^	25.3 (22–28)	25.05 (21.7–28.0)	25.6 (23.3–28.1)	0.4160
Medications				
β‐Blocker before ECV, *n* (%)	78 (40.0%)	37 (37.0%)	41 (43.2%)	0.3803
Amiodarone before ECV, *n* (%)	65 (33.3%)	31 (31.0%)	34 (35.8%)	0.4782
Other antiarrhythmic drugs before ECV, *n* (%)	26 (13.3%)	12 (12.0%)	14 (14.7%)	0.5741
Amiodarone added after ECV, *n* (%)	21 (10.8%)	11 (11.0%)	10 (10.5%)	0.9151
Additional antiarrhythmic drugs after ECV (excluding amiodarone), *n* (%)	20 (10.3%)	10 (10.0%)	10 (10.5%)	0.9036

*Note:* Data are presented as median (interquartile range) for continuous variables and number (percentage) for categorical variables. Comparisons between groups were performed using the Wilcoxon rank‐sum test for continuous variables and the chi‐squared test or Fisher's exact test for categorical variables, as appropriate. *p*‐values < 0.05 were considered statistically significant. ECG composite score was defined as the sum of fragmented QRS, notched P wave, and early repolarization pattern (range, 0–3).

Abbreviations: AF = atrial fibrillation, ECG = electrocardiogram, ECV = electrical cardioversion, ERP = early repolarization pattern, fQRS = fragmented QRS, IQR = interquartile range.

* *p* < 0.05.

** *p* < 0.01.

### 3.2 Electrocardiographic Findings

Electrocardiographic abnormalities were more frequent in patients with early recurrence. The prevalence of notched P‐wave (76.0% vs. 24.2%, *p* < 0.001), fQRS (74.0% vs. 57.9%, *p* = 0.017), and ERP (41.0% vs. 24.2%, *p* = 0.015) was significantly higher in patients with recurrence. The total number of leads with fQRS was also greater in the recurrence group (2 [2–4] vs. 2 [0–3], *p* = 0.029).

Combined ECG abnormalities, including ERP + fQRS (26.0% vs. 10.5%, *p* = 0.006), ERP + notched P‐wave (30.0% vs. 4.2%, *p* < 0.001), and notched P‐wave + fQRS (55.0% vs. 19.0%, *p* < 0.001), were also more common in patients with recurrence. The ECG composite score was significantly higher in the recurrence group (2.0 [1.3–2.0] vs. 1.0 [1.0–2.0], *p* < 0.001). In contrast, PR interval, QRS duration, QTc interval, and other P‐wave indices did not differ significantly between groups.

Intraobserver agreement for ECG interpretation was substantial to almost perfect, with Cohen's *κ* values of 0.93 for notched P‐wave, 0.84 for fQRS, and 0.89 for ERP. The corresponding overall agreement rates were 96.6%, 93.1%, and 96.6%, respectively. Interobserver agreement was also substantial to almost perfect, with Cohen's *κ* values of 0.74 for notched P‐wave, 0.90 for fQRS, and 0.83 for ERP. The corresponding overall agreement rates were 87.8%, 95.9%, and 93.9%, respectively.

### 3.3 Clinical Outcomes

During the 10‐day follow‐up period, early AF recurrence occurred in 100 patients (51.3%). Use of amiodarone or antiarrhythmic drugs after ECV was not significantly associated with recurrence.

### 3.4 Predictors of Early AF Recurrence

**FIGURE 3:**
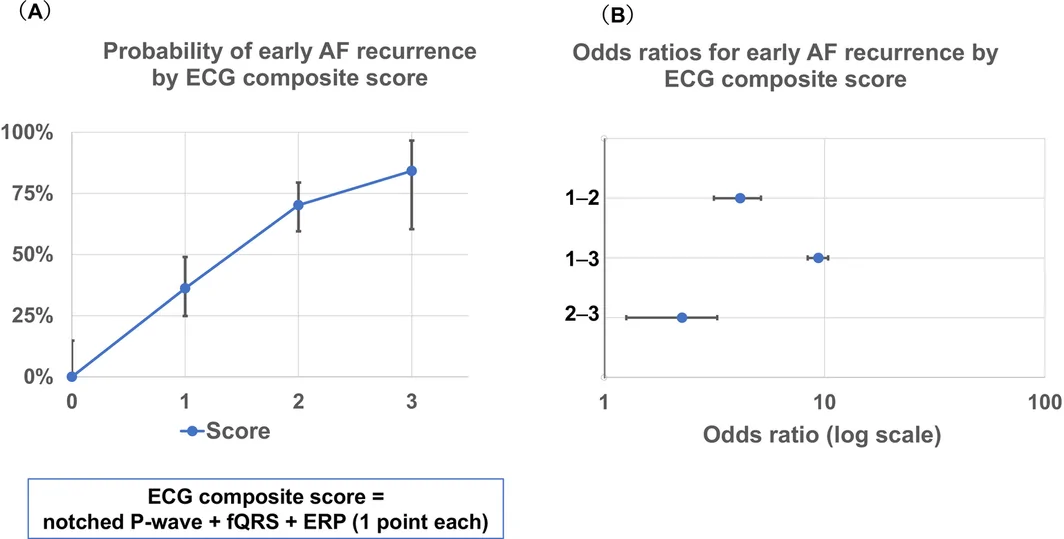
Early atrial fibrillation recurrence according to ECG composite score. (A) Probability of early atrial fibrillation (AF) recurrence within 10 days after electrical cardioversion (ECV) according to ECG composite score categories (0–3). The ECG composite score was defined as the sum of the presence of notched P‐wave, fragmented QRS (fQRS), and early repolarization pattern (ERP), with 1 point assigned for each marker. The probability of recurrence increased stepwise with increasing ECG composite score (*p* < 0.0001). (B) Forest plot showing odds ratios (ORs) with 95% confidence intervals for early AF recurrence in pairwise comparisons of ECG composite score categories (1–2, 1–3, and 2–3). ORs are presented on a logarithmic scale, and the vertical line indicates the reference value of OR = 1. AF, atrial fibrillation; ECG, electrocardiogram; ECV, electrical cardioversion; ERP, early repolarization pattern; fQRS, fragmented QRS.

In univariate logistic regression analysis, AF duration, notched P‐wave, fQRS, ERP, and the ECG composite score were significantly associated with early AF recurrence (Table 2). In multivariable analysis (Model 1), notched P‐wave (OR 11.80, 95% CI 5.51–25.29, *p* < 0.001), fQRS (OR 2.34, 95% CI 1.08–5.07, *p* = 0.031), and ERP (OR 3.54, 95% CI 1.59–7.90, *p* = 0.002) remained independent predictors (Table 3). In Model 2, the ECG composite score remained a strong independent predictor of recurrence (OR 4.91 per 1‐point increase, 95% CI 2.87–8.40, *p* < 0.001), together with AF duration (OR 1.01 per month increase, 95% CI 1.00–1.02, *p* = 0.011). Recurrence rates increased stepwise according to the ECG composite score categories (Figure 3).

**TABLE 2:** Univariate logistic regression analysis of AF recurrence within 10 days.

	Univariate Odds ratio	95% CI	*p*
Age (1 year increase)	1.00	0.97–1.02	0.8361
Male (vs. female)	1.21	0.61–2.38	0.5921
AF duration (1 month increase)	1.01	1.00–1.02	0.0003**
Notched P‐wave	9.91	5.14–19.11	< 0.0001**
Fragmented QRS (Das et al. 2006, 2007)	2.07	1.13–3.79	0.0184*
ERP	2.17	1.18‐4.03	0.0134*
ECG composite score (1 point increase)	4.72	2.85–7.82	< 0.0001**

*Note:* Univariate logistic regression analysis was performed to identify determinants of early recurrence of AF. Odds ratios and the 95% confidence intervals were calculated. ECG composite score was defined as the sum of fragmented QRS, notched P wave, and early repolarization (range, 0–3).

Abbreviations: AF = atrial fibrillation; ECG = electrocardiogram; ERP = early repolarization pattern.

* *p* < 0.05.

** *p* < 0.01.

**TABLE 3:** Multivariable logistic regression analysis of AF recurrence within 10 days.

	Model 1			Model 2		
	Odds ratio	95% CI	*p*	Odds ratio	95% CI	*p*
Age (1 year increase)	1.03	0.99–1.06	0.2973	1.02	0.99–1.05	0.2973
Male (vs. female)	0.51	0.21–1.28	0.1885	0.60	0.23–1.34	0.1885
AF duration (1 month increase)	1.01	1.00–1.01	0.0455*	1.01	1.00‐1.02	0.0114*
Notched P‐wave	11.80	5.51–25.29	< 0.0001**			
Fragmented QRS (Das et al. 2006, 2007)	2.34	1.08–5.07	0.0311*			
ERP	3.54	1.59–7.90	0.0020**			
ECG composite score (1 point increase)				4.91	2.87–8.40	< 0.0001**

*Note:* Multivariable logistic regression analyses were performed to identify determinants of early AF recurrence. Model 1 included age, sex, AF duration, fragmented QRS, early repolarization, and notched P‐wave. Model 2 included age, sex, AF duration, and ECG composite score. The ECG composite score was defined as the sum of fragmented QRS, notched P‐wave, and early repolarization pattern (range, 0–3). Odds ratios and the 95% confidence interval were calculated.

Abbreviations: AF = atrial fibrillation, CI = confidence interval, ECG = electrocardiogram, ERP = early repolarization pattern.

* *p* < 0.05.

** *p* < 0.01.

A supplementary analysis using a regression coefficient‐based weighted ECG score was performed. Based on the relative magnitude of the regression coefficients, we developed a simplified weighted ECG score by assigning integer values of 3, 1, and 2 points to notched P‐wave, fQRS, and ERP, respectively. The weighted ECG score was significantly associated with AF recurrence within 10 days after cardioversion, with an OR of 2.11 per 1‐point increase (95% CI, 1.70–2.61; *p* < 0.001). The AUC of the weighted ECG score was 0.813, compared with 0.770 for the original equal‐weight ECG score. At the optimal cutoff, the weighted ECG score yielded a sensitivity of 84.0% and specificity of 67.4%, whereas the equal‐weight ECG score yielded a sensitivity of 75.0% and specificity of 70.5% (Table S1).

## 4 Discussion

### 4.1 Major Findings

The principal findings of this study were as follows. First, notched P‐wave, fQRS, and ERP on a standard 12‐lead ECG obtained immediately after successful ECV were significantly associated with early recurrence of AF. Second, these ECG markers remained independent predictors after multivariable adjustment. Third, the ECG composite score integrating these three findings showed a stepwise association with recurrence risk and provided simple risk stratification after ECV. These findings suggest that surface ECG may capture underlying electrophysiological and structural substrates associated with early AF recurrence.

In contrast to many previous studies, left atrial diameter (LAD) was not significantly associated with early AF recurrence in the present study. This discrepancy may be partly explained by our short‐term endpoint, which focused on recurrence detected at the scheduled 10‐day follow‐up ECG, whereas many prior studies evaluated longer‐term recurrence after cardioversion. In addition, LAD is a simple anteroposterior linear measurement and may not fully reflect asymmetric left atrial remodeling or three‐dimensional atrial enlargement. Left atrial volume or left atrial volume index may better capture structural atrial remodeling and may therefore provide stronger prognostic information.

### 4.2 Clinical Significance of Early Recurrence After ECV

Although catheter ablation has become an established rhythm‐control strategy (Andrade et al. 2021; Kirchhof et al. 2020), ECV remains an important therapeutic option in patients with persistent AF. Restoration of sinus rhythm by ECV may help clarify symptom burden (Ganapathy et al. 2015), assess sinus node function (Kamada et al. 2022), and determine whether maintenance of sinus rhythm is clinically beneficial. In some patients who appear asymptomatic during AF, symptom improvement after restoration of sinus rhythm may reveal previously unrecognized AF‐related symptoms. In addition, response to ECV may help guide subsequent management, including the timing and indication for catheter ablation. However, because early AF recurrence is common, better selection of patients who are likely to maintain sinus rhythm is clinically desirable.

### 4.3 Notched P‐Wave

The P‐wave reflects atrial depolarization and conduction (De Bacquer et al. 2007). A notched P‐wave is considered a marker of intra‐ and interatrial conduction delay (Dilaveris et al. 1998), which may result from atrial remodeling, fibrosis, and impaired conduction through Bachmann's bundle or adjacent septal pathways. Previous studies (Okuyama et al. 2024) have linked abnormal P‐wave morphology to incident AF and recurrence after catheter ablation. Our findings extend these observations by demonstrating that notched P‐wave was strongly associated with early recurrence after ECV. This suggests that restoration of sinus rhythm does not eliminate the underlying atrial substrate, and residual conduction abnormalities may facilitate rapid AF reinitiation.

### 4.4 fQRS

fQRS has been recognized as a marker of conduction heterogeneity, myocardial scar, and structural abnormalities (Das et al. 2007). Although traditionally linked to ventricular arrhythmogenic substrate, previous studies (Eren et al. 2020) reported an association between fQRS and AF recurrence after ECV, and our findings are consistent with those observations. In the present study, however, neither the overall distribution of underlying cardiac disease nor the prevalence of heart failure differed significantly between patients with and without early AF recurrence. Therefore, the association between fQRS and AF recurrence cannot be explained simply by differences in clinically apparent structural heart disease or heart failure. Given that fQRS is not a direct marker of atrial pathology, the mechanism underlying this association remains uncertain and requires further investigation.

### 4.5 ERP

ERP has been associated with ventricular arrhythmias, sudden cardiac death, and increased electrical vulnerability (Macfarlane et al. 2015). Emerging evidence also suggests a relationship between ERP and AF occurrence or recurrence after catheter ablation (Hasegawa et al. 2019; Hunuk et al. 2019). Our results support the concept that ERP may represent a broader electrophysiological phenotype characterized by both atrial and ventricular susceptibility to arrhythmia. Although the precise mechanism remains uncertain, autonomic influences and repolarization heterogeneity may contribute.

### 4.6 Value of the ECG Composite Score

An important finding of the present study is the strong predictive value of the ECG composite score. Because each component can be assessed from a routine 12‐lead ECG without additional cost or specialized equipment, this score may be particularly useful in daily clinical practice. The stepwise increase in recurrence across score categories suggests incremental risk stratification and may help identify patients who require closer follow‐up, early antiarrhythmic intervention, or earlier referral for catheter ablation.

In a supplementary analysis, a regression coefficient‐based weighted ECG score showed numerically better discrimination than the equal‐weight ECG composite score. However, we retained the equal‐weight ECG composite score as the primary score because it is simpler, easier to interpret, and may be more practical for clinical use. In addition, because the weighted ECG score was derived from the present cohort and has not been externally validated, it may be more susceptible to overfitting. Further studies are needed to validate the weighted ECG score and confirm its generalizability.

### 4.7 Clinical Implications

These findings indicate that simple ECG markers obtained immediately after successful ECV may help identify patients at high risk of early AF recurrence. Because these parameters are inexpensive, widely available, and noninvasive, they may facilitate bedside risk stratification and guide postcardioversion management, including closer rhythm monitoring, early follow‐up, consideration of antiarrhythmic therapy, or earlier referral for catheter ablation.

However, the timing of ECG assessment should be considered when interpreting these findings. ECV itself may transiently alter atrial electrophysiological properties after restoration of sinus rhythm. Previous studies have shown that atrial refractoriness and conduction properties may change dynamically after cardioversion, reflecting recovery from AF‐induced electrical remodeling (Sato et al. 2001; Yu et al. 1999). Therefore, ECG markers assessed immediately after cardioversion may reflect not only the underlying atrial substrate but also acute postcardioversion electrophysiological changes. Accordingly, the predictive value of these ECG markers should be interpreted with this potential influence in mind.

### 4.8 Study Limitations

This study has several limitations. First, this was a retrospective single‐center study with a modest sample size, which may limit the generalizability of the findings. Second, continuous rhythm monitoring was performed only during hospitalization and was not continued after discharge. Therefore, asymptomatic or transient AF recurrence before the scheduled 10‐day follow‐up visit may have been underdetected, and ascertainment bias related to the intensity and duration of rhythm monitoring cannot be excluded. Third, because ECG markers were assessed immediately after ECV, we cannot completely exclude the possibility that acute postcardioversion electrophysiological changes, electrical stunning, the number of shocks delivered, cumulative energy, sedation, or concomitant antiarrhythmic drug therapy influenced ECG parameters. These factors were not fully accounted for in the present analysis and should be considered when interpreting the results. Fourth, although left atrial volume index may better reflect left atrial remodeling than LAD, it was not consistently available throughout the entire study period. Therefore, LAD, which was available for the entire cohort, was used in the main analysis. Finally, external validation of the ECG composite score, including the weighted ECG score evaluated in the supplementary analysis, was not performed. Prospective multicenter studies with standardized rhythm monitoring are warranted to validate these findings.

## 5 Conclusions

Notched P‐wave, fQRS, and ERP on a standard 12‐lead ECG were independently associated with early AF recurrence after ECV in patients with persistent AF. An ECG composite score based on these markers may provide a simple and practical tool for post‐ECV risk stratification.

## Author Contributions

Keisuke Yonezu acquired, analyzed, and interpreted the data and drafted the manuscript. Hirochika Yamasaki, Kei Hirota, Hidekazu Kondo, Akira Fukui, Hidefumi Akioka, and Yasushi Teshima contributed to data acquisition, data analysis, and interpretation of the results. Tetsuji Shinohara and Naohiko Takahashi conceived and designed the study and supervised the research. All authors critically reviewed the manuscript, approved the final version, and agreed to be accountable for all aspects of the work.

## Funding

The authors have nothing to report.

## Disclosure

The authors have nothing to report.

## Conflicts of Interest

The authors declare no conflicts of interest.

## Supporting information


**Table S1:** Comparison of equal‐weight and weighted ECG scores for predicting early AF recurrence.

## Data Availability

The data that support the findings of this study are available from the corresponding author upon reasonable request.
