# Supplementary material for: Electrocardiographic Predictors of Early Recurrence of Atrial Fibrillation After Electrical Cardioversion
Source: Ann Noninvasive Electrocardiol. 2026 Aug 28;31(5):e70225. doi: 10.1111/anec.70225 (PMC13523162; doi:10.1111/anec.70225)
Supplement: Supplementary file 1 [file ANEC-31-e70225-s001.docx]

Supplementary Table S1. Comparison of equal-weight and weighted ECG scores for predicting early AF recurrence

| Score | OR per 1-point increase | 95% CI | p value | AUC | Optimal cutoff | Sensitivity | Specificity |
| --- | --- | --- | --- | --- | --- | --- | --- |
| Equal-weight ECG score | 4.91 | 2.91–7.82 | <0.0001 | 0.770 | ≥2 | 75.0% | 70.5% |
| Weighted ECG score | 2.11 | 1.70–2.61 | <0.0001 | 0.813 | ≥3 | 84.0% | 67.4% |

P values <0.05 were considered statistically significant.

ECG composite score was defined as the sum of fragmented QRS, notched P wave, and early repolarization pattern (range, 0–3).

AUC = area under the curve, CI = confidence interval, ECG = electrocardiogram, OR = odds ratio.
